# Integrated Lower Limb Robotic Orthosis with Embedded Highly Oriented Electrospinning Sensors by Fuzzy Logic-Based Gait Phase Detection and Motion Control

**DOI:** 10.3390/s25051606

**Published:** 2025-03-05

**Authors:** Ming-Chan Lee, Cheng-Tang Pan, Jhih-Syuan Huang, Zheng-Yu Hoe, Yeong-Maw Hwang

**Affiliations:** 1 Department of Electrical Engineering, National Kaohsiung University of Science and Technology, Kaohsiung 807, Taiwan; mclee@nkust.edu.tw; 2 Department of Mechanical and Electro-Mechanical Engineering, National Sun Yat-sen University, Kaohsiung 804, Taiwan; pan@mem.nsysu.edu.tw (C.-T.P.); andyhuang@mem.nsysu.edu.tw (J.-S.H.); ymhwang@mail.nsysu.edu.tw (Y.-M.H.); 3 Institute of Advanced Semiconductor Packaging and Testing, College of Semiconductor and Advanced Technology Research, National Sun Yat-sen University, Kaohsiung 804, Taiwan; 4 Institute of Precision Medicine, National Sun Yat-sen University, Kaohsiung 804, Taiwan; 5 Taiwan Instrument Research Institute, National Applied Research Laboratories, Hsinchu City 300, Taiwan; 6 Department of Physical Medicine and Rehabilitation, Kaohsiung Veterans General Hospital, Kaohsiung 813, Taiwan; 7 Department of Pharmacy and Master Program, Tajen University, Pingtung County 90741, Taiwan

**Keywords:** exoskeleton robot, gait phase, ground reaction force, near-field electrospinning, fuzzy logic, master–slave control

## Abstract

This study introduces an integrated lower limb robotic orthosis with near-field electrospinning (NFES) piezoelectric sensors and a fuzzy logic-based gait phase detection system to enhance mobility assistance and rehabilitation. The exoskeleton incorporates embedded pressure sensors within the insoles to capture ground reaction forces (GRFs) in real-time. A fuzzy logic inference system processes these signals, classifying gait phases such as stance, initial contact, mid-stance, and pre-swing. The NFES technique enables the fabrication of highly oriented nanofibers, improving sensor sensitivity and reliability. The system employs a master–slave control framework. A Texas Instruments (TI) TMS320F28069 microcontroller (Texas Instruments, Dallas, TX, USA) processes gait data and transmits actuation commands to motors and harmonic drives at the hip and knee joints. The control strategy follows a three-loop methodology, ensuring stable operation. Experimental validation assesses the system’s accuracy under various conditions, including no-load and loaded scenarios. Results demonstrate that the exoskeleton accurately detects gait phases, achieving a maximum tracking error of 4.23% in an 8-s gait cycle under no-load conditions and 4.34% when tested with a 68 kg user. Faster motion cycles introduce a maximum error of 6.79% for a 3-s gait cycle, confirming the system’s adaptability to dynamic walking conditions. These findings highlight the effectiveness of the developed exoskeleton in interpreting human motion intentions, positioning it as a promising solution for wearable rehabilitation and mobility assistance.

## 1. Introduction

Exoskeleton robots are gaining attention due to advancements in personal care and nursing robots, driven by the rising demand for assistive orthoses and concerns over an aging population. According to United Nations population statistics [1], the aging population continues to grow, increasing the demand for healthcare support. The development of assistive technologies has become essential to alleviate the strain on personal care services. In the 1960s, General Electric Company designed the first exoskeleton, Hardiman, which utilized motors and a master–slave control system. However, its complex and heavy structure prevented successful operation [2]. Later, researchers at Tsukuba University developed the Hybrid Assistive Limb (HAL) to assist patients with lower-limb disorders. This system used sensors to capture foot reaction forces and detect the user’s motion intention [3]. The Berkeley Lower-Extremity Exoskeleton (BLEEX) incorporated seven degrees of freedom (DoFs) per leg, with four powered by linear hydraulic actuators. BLEEX allowed users to carry loads effortlessly across various terrains [4,5].

Recent advancements in sensors have accelerated the development of robotic exoskeletons. Signor et al. [6] leveraged magnetic force sensors to create a force-sensing prototype for robotic applications. Orekhov et al. [7] designed an ankle exoskeleton with a parallel elastic element, optimizing a controller to maximize its benefits. Xue et al. [8] introduced a wearable robotic hip exoskeleton powered by two electrical series elastic actuators to assist elderly and disabled individuals. Shushtari et al. [9] proposed a novel method for reference trajectory adaptation in lower-limb rehabilitation exoskeletons, ensuring adjustments based on a patient’s motor capacity. Qian et al. [10] developed a sensor-based locomotion mode recognition system and an adaptive gait phase estimation approach to enable terrain-adaptive assistive walking. Furukawa et al. [11] incorporated electromyography (EMG) movement classification to develop a lightweight knee exoskeleton using a carbon fiber frame and a pneumatic artificial muscle-driven joint.

Khazoom et al. [12] designed an exoskeleton controlled by a state map controller to provide real-time assistance for walking, jumping, and landing. Sanz-Merodio et al. [13] introduced a lower-limb exoskeleton that enabled basic standing, sitting, and walking motions, integrating a compliance controller that utilized insole sensor measurements. Matsubara et al. [14] proposed an adaptive walking assistance exoskeleton, evaluating it through simulated user and exoskeleton models. Kim et al. [15] developed a hydraulic lower extremity exoskeleton with dual-mode control, featuring an active mode for the stance phase and a passive mode for the swing phase.

For gait phase detection [16], Srivises et al. [17] designed a fuzzy logic-based smart shoe that used four force sensors on the insole to analyze gait—a fuzzy logic algorithm determined real-time gait phases such as stance, heel-off, swing, and heel-strike. Xu et al. [18] developed a fuzzy logic method to classify human gait phases based on foot pressure data, selecting sensor positions based on plantar pressure distributions. Ding et al. [19] introduced a proportion-based fuzzy algorithm and designed a smart insole with embedded pressure sensors for improved detection adaptability. Song et al. [20] implemented a fuzzy logic-based method for controlling a lower-limb exoskeleton robot, adjusting the hydraulic cylinder’s stroke based on hip angle and gait phase.

Advancements in motor control have further improved exoskeleton performance. Tang et al. [21] developed an optimal fuzzy proportional-integral-derivative (PID) controller with adaptive capabilities. Jiao et al. [22] applied a genetic algorithm to optimize a self-adaptive sliding mode position controller, demonstrating improved control performance. Jafari et al. [23] explored recurrent neural networks (RNNs) for nonlinear system control, creating an RNN-based adaptive PID controller. Hamouda et al. [24] combined an adaptive neuro-fuzzy inference system (ANFIS) with particle swarm optimization (PSO) to enhance controller performance. Zhu et al. [25] integrated lower limb exoskeletons with three-dimensional force sensors, employing a PID controller to adjust movement based on wearer intention recognition. Chen et al. [26] developed a control strategy to manage multiple walking phases, including single-leg and double-leg support, using a torque allocation method and force controller for robust interaction force control. These studies emphasize integrating sensors and controllers into exoskeletons to enhance user experience [27].

Electrospinning technology provides a simple yet effective method for fabricating polymer materials into nanometer-scale fibers [28]. This process relies on electrostatic forces to manipulate polymer solutions. Far-field electrospinning (FFES) occurs when the electric field distance exceeds 10 cm. In conventional electrospinning, a constant-flow pump delivers a polymer solution through a syringe, forming a droplet at the needle tip. When a high-voltage electric field is applied, the solution becomes charged, accumulating surface charges. As the electric field strength increases, repulsive forces overcome surface tension, elongating the droplet into a Taylor cone. Further increases in electric field strength cause the charged polymer solution to eject from the cone tip, forming fine fibers that deposit onto a collector [29].

He et al. [30] introduced near-field electrospinning (NFES), which enables precise fiber deposition while reducing spinning voltage. Unlike traditional electrospinning, which produces randomly distributed fibers, NFES ensures controlled positioning, making it ideal for electronics, energy harvesting, flexible sensors, and tissue engineering applications. Despite challenges in large-scale fiber production and fiber diameter consistency, NFES holds significant potential for micro/nanofiber fabrication. Du et al. [31] developed a novel adaptive wheel motion generator for a wheeled quadruped robot that leverages centroidal dynamics to track the robot’s centroidal motion. This generator combines a whole-body inverse kinematics model with a centroidal momentum/dynamics model, allowing the robot to adapt to rough terrains. Key aspects of the innovation include adaptive motion generation, a precise and robust integration of centroidal dynamics, and a prioritized impedance controller to ensure stability and compliance during locomotion. Xu et al. [32] presents a novel horizon-stability control framework for wheel-legged hybrid robots to maintain a stable and horizontal trunk while traversing unknown, rough terrains. The framework consists of a compliance controller and a terrain adaptation controller to address challenges such as multi-end-effectors control coupling (MEC) and multi-task force-tracking control (MFTC). The compliance controller leverages adaptive impedance control to ensure compliant interactions with the terrain and track desired ground reaction forces, while the terrain adaptation controller decouples posture adjustments to regulate control outputs in response to terrain changes. Table 1 lists recent research on exoskeletons.

**Figure 1:**
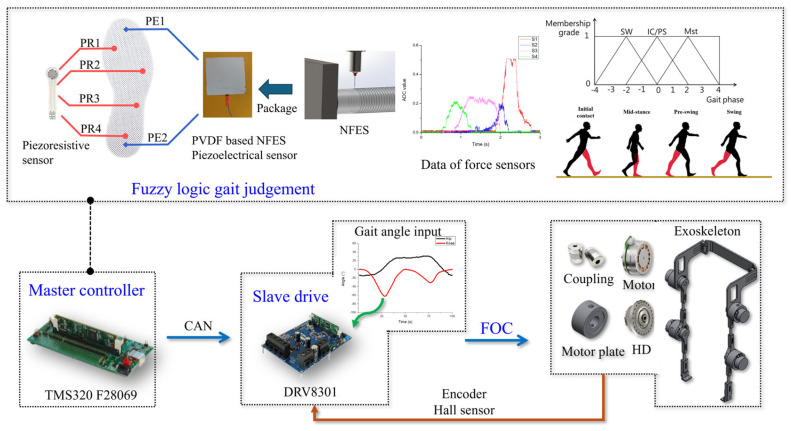
The experimental process of this integrated system in this study.

This study presents a fuzzy logic-based gait phase detection system integrated with a lower limb exoskeleton robot. The system incorporates PVDF-based NFES piezoelectric sensors on insoles. Measurements determined the optimal positions for four force-sensing resistors. The signal acquisition system includes a Texas Instruments (TI) microcontroller, sensors, and peripherals. The insole sensor array captures ground reaction forces while the wearer walks. Analyzed gait phase and motion intention data are processed and defuzzified by the master controller before being transmitted to the exoskeleton’s slave controller. The slave controller then executes the appropriate gait pattern and actuates the motors at the joints. Figure 1 provides the experimental process of this integrated system in this study. The key contributions of this study are as follows:

(1) Development of a lower limb robotic orthosis: A lightweight and safety-conscious exoskeleton was designed to measure ground reaction forces and accurately detect gait phases.
(2) Innovative human–machine interaction sensor: A novel NFES-based piezoelectric sensor was designed and fabricated, offering high flexibility and sensitivity, focusing on tracking the center of body pressure.
(3) Fuzzy logic-based gait phase detection: Implementing and designing a customized fuzzy logic algorithm, well-defined fuzzy rules, and membership functions to enhance gait phase recognition accuracy.
(4) Experimental validation of motion control: Motion control experiments were conducted across different walking cycle periods, demonstrating the reliability and effectiveness of the master–slave control framework.

## 2. Method

### 2.1. Robotic Orthosis with the Signal Acquisition System

In this study, we designed and developed a lower-limb exoskeleton robot to assist the wearer in walking. Motors and harmonic drives (HDs) were installed at the hip and knee joints to control the movement of the leg rods. The exoskeleton robot featured eight degrees of freedom (DOFs), with each leg incorporating four DOFs at the waist, hip, knee, and ankle. The design closely followed the natural DOFs of human legs, ensuring smooth and natural movement. The two rotational axes at the waist improved wearer comfort, while the hip and knee joints facilitated joint movements necessary for walking.

The exoskeleton robot’s mechanical design, stress simulation, and gait analysis were created using the 3D modeling software SOLIDWORKS 2023 (3D Systems Inc., Rock Hill, SC, USA). The exoskeleton was manufactured with computerized numerical control (CNC) machining, ensuring high precision. Aluminum 6061 alloys (Al-6061) were used for the structure, offering advantages such as lightweight properties, ductility, and ease of machining. The total weight of the exoskeleton, including motors and HDs, was approximately 16 kg.

**Figure 2:**
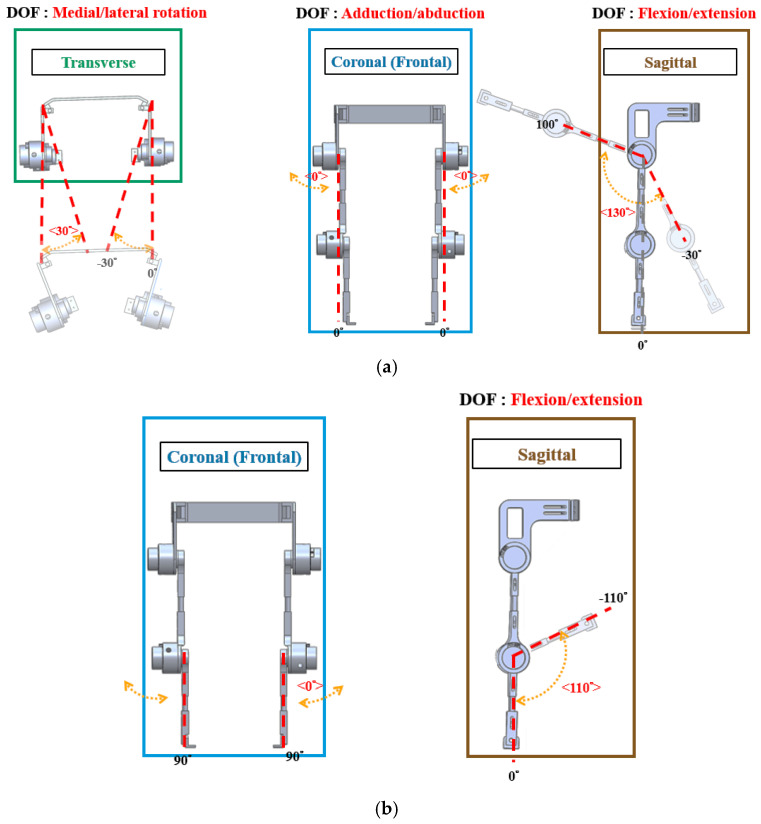
Rotation angles and DOFs of the robotic orthosis. (**a**) Range of motion of the hip (**b**) Range of motion of the knee.

To accommodate wearers of different heights, the exoskeleton featured an adjustable-length design. Adjustable lower-limb components connected the joints. As shown in Figure 2a,b, the hip joint moved in the sagittal plane with one DOF, supporting flexion and extension to propel the wearer forward. The design excluded adduction/abduction DOFs to maintain balance and stability, while medial/lateral rotation in the transverse plane enhanced mobility. The knee joint allows flexion and extension in the sagittal plane, enabling the knee to lift the foot off the ground and provide support while standing. The ankle joint had one DOF for plantar flexion/dorsiflexion in the sagittal plane, ensuring overall stability.

**Figure 3:**
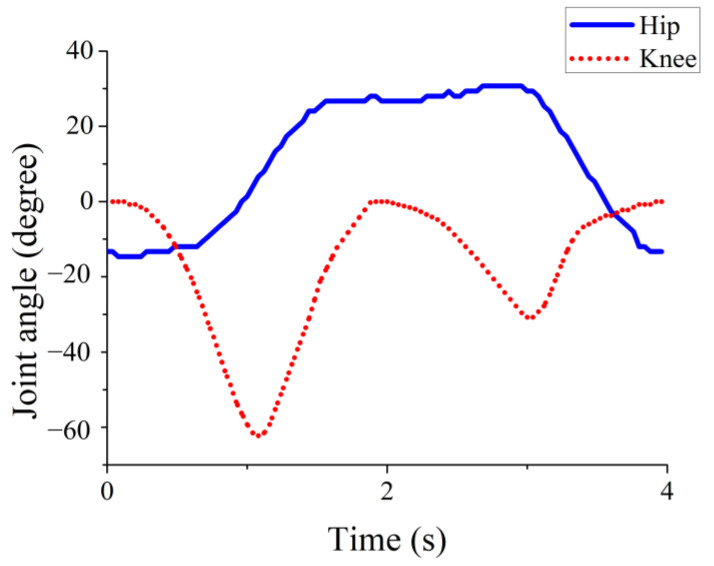
Gaits of the hip and knee.

A gait experiment was conducted to analyze both the wearer’s gait and the robotic orthosis movement. Figure 3 illustrates the analysis of the hip and knee joint motion during walking.

The signal acquisition system comprised a microcontroller and resistive pressure sensors. Sensors captured ground reaction forces, and the microcontroller processed the force signals and calculations. The TMS320F28069 Piccolo controller (Texas Instruments, Dallas, TX, USA) was used to develop the fuzzy logic system and receive and analyze the force signals of each sensor point on the insole. An analog-to-digital converter (ADC) was used on the controller to receive values. We choose the typical 12-bit ADC, which ranges from 0 to 4095. The master controller sent the gait phase information to the slave controller and could also receive feedback from the slave controller to fine-tune the adaptability. The slave controller was the TI DRV8301/TMS320F28069 (Texas Instruments, Dallas, TX, USA) evaluation board, which controlled the joint drive of the exoskeleton robot and was responsible for receiving the gait phase information sent by the master controller.

**Figure 4:**
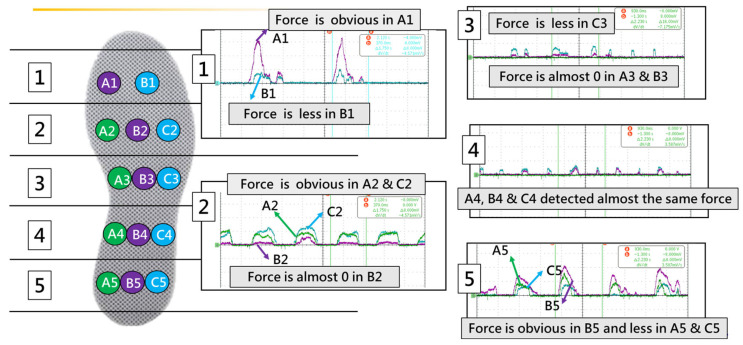
Tests of the possible stress points of the feet.

**Figure 5:**
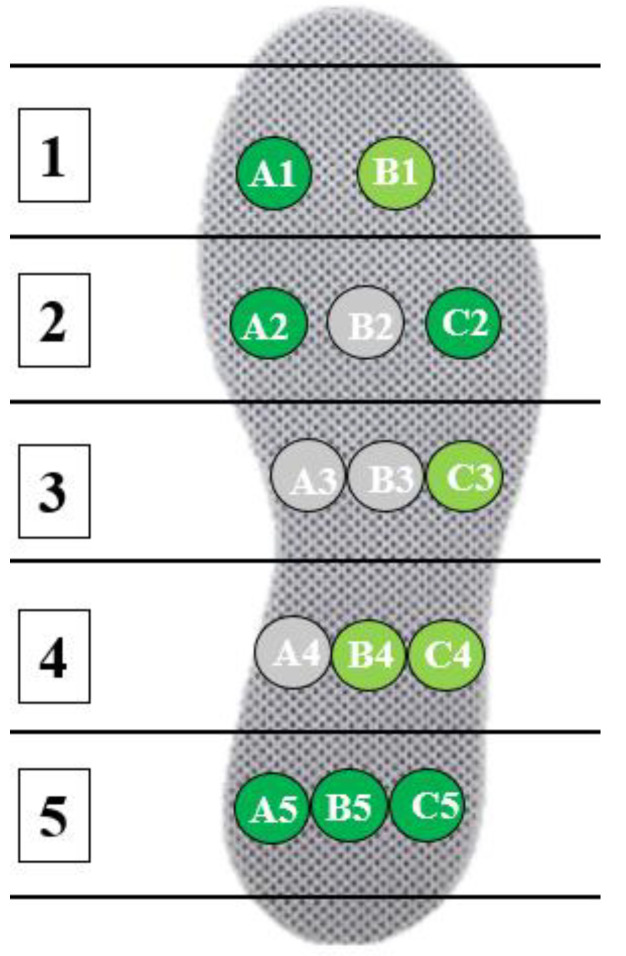
Stress points of the feet.

In addition, to verify the maximum force-bearing position when the subject wore the insole, the gait cycle was divided into four main intervals. Two main stages: the mid-stance phase (Mst), when the foot is entirely on the ground, and the swing phase (SW), when the foot is entirely off the ground. There are two transition phases: initial contact (IC) with the heel strikes and pre-swing (PS) with only the toe strikes. The decision to place the sensors was based on the possible stress points of the feet in each gait. Multiple resistive sensors (A1 to A5, B1 to B5, and C2 to C5) were placed on the insole in a horizontal arrangement (five horizontal areas). Several complete gait cycles stepped sequentially to obtain force signals at each point, as shown in Figure 4. As shown in Figure 5, dark green points were the feet’s stress points.

**Figure 6:**
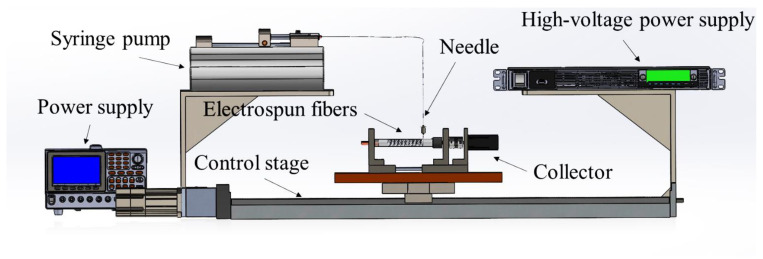
Schematic of NFES.

**Figure 7:**
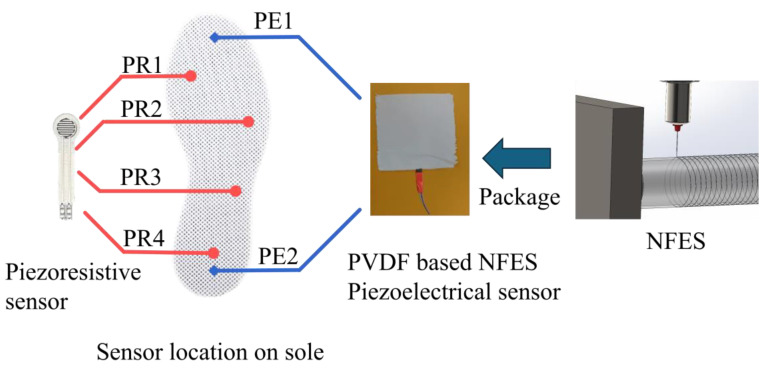
Positions of the sensors on the insole.

NFES is an advanced micro/nanofabrication technique that enhances traditional electrospinning by enabling precise fiber deposition. Figure 6 illustrates the NFES setup, which consists of a high-voltage power supply, a syringe pump, a control stage, and a collector. By applying a high-voltage electric field, the system draws polymer solutions into ultrafine fibers and deposits them in controlled patterns on substrates positioned just 1–3 mm from the spinneret, ensuring high accuracy and proper alignment. To optimize signal computation efficiency and maintain device reliability when multiple sensors were integrated into the insole, only four strategic points were selected for placing the resistive sensors. Figure 7 shows the sensor placements on the insole, where PE represents the PVDF-based NFES piezoelectric sensor and PR denotes the piezoresistive sensor.

### 2.2. Design of a Fuzzy Logic-Based Gait Phase Strategy for Robotic Orthosis

**Figure 8:**
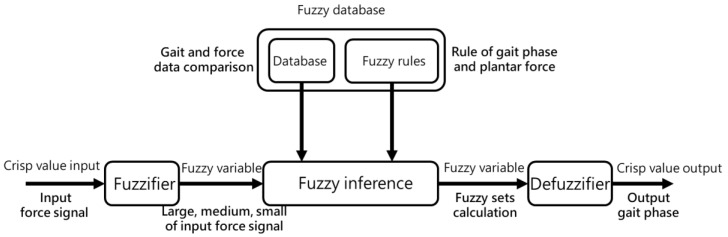
Fuzzy logic structure.

Fuzzy logic partitions and simplifies a complex nonlinear system into a multi-segment linear form, allowing for the quick and easy identification of intervals or solutions for each input. It translates various data and collected information into fuzzy expressions, designing fuzzy sets based on comparing input value proportions in each rule. This process ensures accurate and adaptable decision-making. The fuzzy logic system in this study includes crisp value input, fuzzification, fuzzy inference, defuzzification, and crisp value output. Figure 8 illustrates the fuzzy logic structure. The system applies fuzzy function membership to determine fuzzy value inputs in the crisp value input stage. The fuzzifier, fuzzy database, and fuzzy rules then process these inputs, selecting the appropriate fuzzy value outputs for further inference.

The deduction process and design method of the fuzzy logic to detect gait phases in this study were described below:

(1) Input the ground reaction force signals of the front and rear pressure groups on the insole.
(2) Fuzzifier.
(3) The collected data and the designed fuzzy rules were used to infer the fuzzy membership functions to which the force signal belongs.
(4) Defuzzifier
(5) Output the results of the crisp gait phases.

When the sensor array on the insole was pressured, the system received a combination of signals from each point of the foot pressure sensor array, and the fuzzy membership functions were designed.

**Figure 9:**
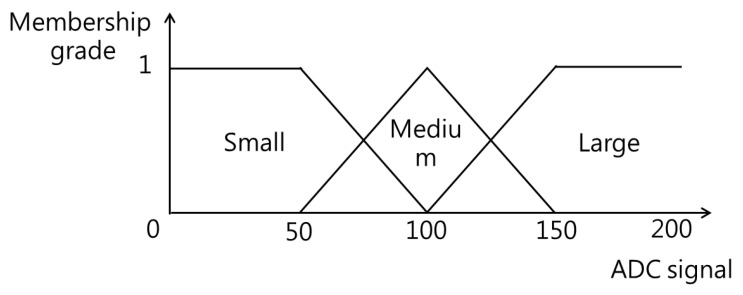
The fuzzy membership functions of ground reaction forces.

The first two sensors comprised the front group, while the last two formed the rear group. Ground reaction forces were similarly classified into these two groups, with pressure values determined by averaging the readings from the corresponding sensors in each group. The fuzzy membership functions for ground reaction forces were defined across three semantic intervals: “small” (ADC value: 0 to 100), “medium” (ADC value: 50 to 150), and “large” (ADC value: above 100). Figure 9 illustrates the triangular membership function, one of the most commonly used functions in fuzzy logic design.

**Figure 10:**
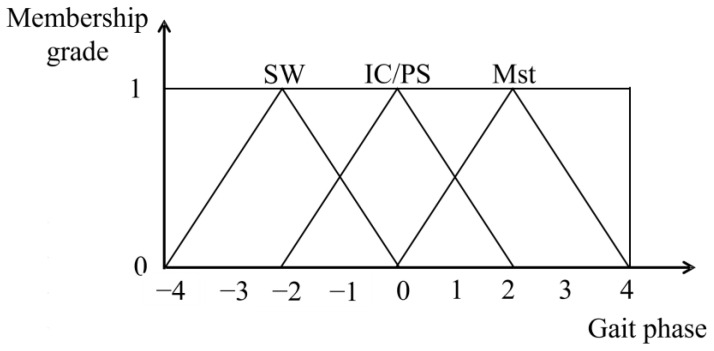
The fuzzy membership functions of gait phases.

The membership function and fuzzy rules of gait phases were designed as shown in Figure 10 and Table 2. The study mainly divided the walking cycle into intervals, which meant the Mst phase, SW phase, and other transition phases of the gait phase. In the transition phases, an additional interval, the PS phase, was designed when Mst moved forward to SW. In addition, the transition period was intended as IC when SW moves forward to Mst.

The fuzzy inference could be executed once the fuzzy memberships for ground reaction forces, gait phases, and fuzzy rules are established. After the fuzzifier processed the inputs, the front and rear groups served as fuzzy input variables, which were then interpreted using the fuzzy membership functions. The semantic intervals were classified as “small”, “medium”, and “large”.

For example, if the input ground reaction forces were [front group, rear group] = [100, 50], the corresponding membership values would be determined as follows:

$$\mu_{Medium}\left(100\right)=1\;\mathrm{for}\;\mathrm{the}\;\mathrm{front}\;\mathrm{group}$$

$$\mu_{Small}\left(50\right)=1\;\mathrm{for}\;\mathrm{the}\;\mathrm{rear}\;\mathrm{group}$$

It could be observed that the membership values of the front group and the rear group were 1 for “medium” and 1 for “small” by the fuzzy inference. That meant the fuzzy logic classified the current states of ground reaction forces. Furthermore, fuzzy rules were taken to determine the gait phase. For example, a fuzzy rule expressed, “If the force of the front group is medium and the force of the rear group is small, the gait phase is PS”, the PS membership grade of this input could be expressed as below:

$$\mu_{\mathrm{PS}}\left(100,\;50\right)=\left(1,1\right)=1$$

**Figure 11:**
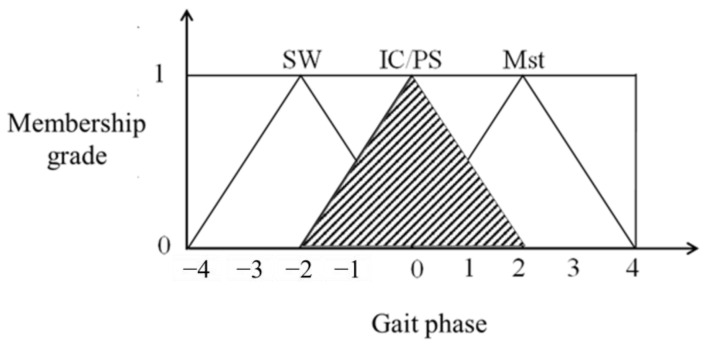
The schematic diagram of the area of fuzzy sets.

Table 2 lists the fuzzy rules used in this study. After searching all fuzzy sets of each gait phase, the area of fuzzy sets could be calculated. Figure 11 shows a schematic diagram of the size of the PS fuzzy sets.

This study’s defuzzification method was the center of gravity (COG) method to output the exact gait phase. The equation of COG is shown in Equation (1). The input values, fuzzy rules, and fuzzy membership functions verified the results of defuzzification.

(1) $$\mu^{*}=\frac{\int_{-2}^{0}\left(\frac{1}{2}u+1\right)udu+\int_{0}^{2}\left(\frac{-1}{2}u+1\right)udu}{\int_{-2}^{0}\left(\frac{1}{2}u+1\right)du+\int_{0}^{2}\left(\frac{-1}{2}u+1\right)du}=0$$

where $\mu^{*}$ was the result of the COG method and u was the fuzzy membership grade.

The result of the fuzzy gait stage was 0. Since the sensor value of the front group was greater than that of the rear group, it was the PS gait stage.

### 2.3. The Proposed Algorithm with the Master-Slave Control System

**Figure 12:**
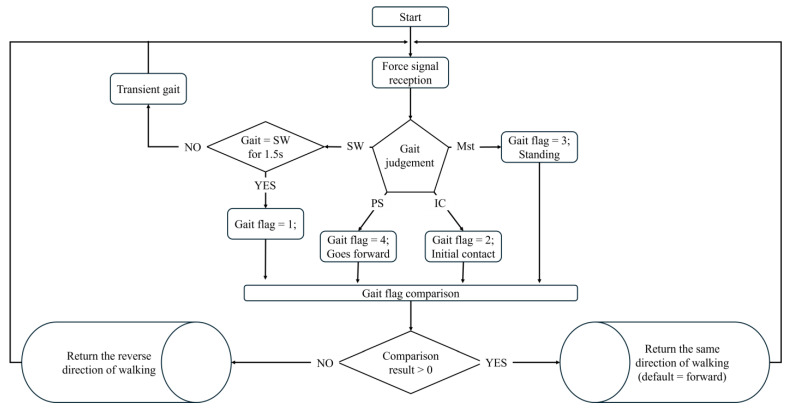
Flowchart of the gait phase detection.

The master-slave control system included a master controller (TI TMS320F28069 Piccolo (Texas Instruments, Dallas, TX, USA)) and four slave controllers (TI DRV8301/TMS320F28069 evaluation boards (Texas Instruments, Dallas, TX, USA)). The master controller handled the processing of ground reaction force signals and fuzzy logic calculations, while the slave controllers managed joint motor control through controller area network (CAN) transmission. Figure 12 illustrates the master controller’s decision-making mechanism and signal transmission process. The system implemented an internal multi-tasking mechanism using multiple interrupt programs.

The system temporarily ignored the SW phase for 1 to 2 s during gait phase transitions for gait phase computation. This adjustment accounted for the inability to achieve seamless connections between gait stages in an ideal cycle diagram (e.g., transitioning from IC to Mst or from Mst to PS). Testing confirmed that the overall decision-making mechanism remained functional, even considering the absolute SW phase.

The system fine-tuned force measurements in the posture learning phase to ensure accurate calibration. When the wearer stood upright and relaxed while wearing the insoles, the system adjusted to establish a standard force value, optimizing the subject’s baseline posture for more precise gait phase detection.

**Figure 13:**
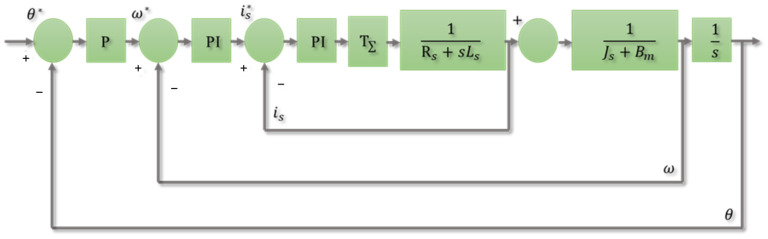
The schematic diagram of the designed three-loop control.

The control system of this study was designed based on the field-oriented control (FOC) three-loop control. The current loop and speed loop were controlled by the proportional-integral (PI) controller, respectively. The proportional (P) controller regulates the parameters of the position loop. The control framework of this study also designed parameters in the controllers, which enabled the system to change the control parameters in real time. The schematic diagram of the designed three-loop control is shown in Figure 13, where $\theta^{*}$, $\omega^{*}$, and ${i_{s}}^{*}$ are the position, voltage, and current commands, respectively, and θ, ω, and $i_{s}$ are the position, voltage, and current feedback values, respectively.

The open–loop transfer function $G_{o,c}$ is expressed as shown in Equation (2) for the current loop. It comprises an electrical motor model and a PI controller.

(2) $$G_{o,c}=k_{p,c}\left(1+\frac{1}{sT_{i,c}}\right)\frac{1}{R_{s}+sL_{s}}e^{-sT_{\sum }}$$

where $k_{p,c}$ and $T_{i,c}$ are the PI controller gain and time constant of the current loop, respectively; $T_{\sum }$ is the delay time; and $R_{s}$ and $L_{s}$ are the resistance and inductance of the motor, respectively.

Then, the speed control loop is modeled. The open-loop transfer function is represented by Equation (3). The speed loop comprises a mechanical motor model and a PI controller. A single delay time, $T_{\sum s}$, is obtained by the dead time and delay time of the speed control loop merged. $k_{p,s}$ and $T_{i,s}$ are the PI controller gain and time constant of the speed loop, respectively.

(3) $$G_{o,s}=K_{p,s}\left(1+\frac{1}{sT_{i,s}}\right)\left(\frac{1}{Js+B_{m}}\right)G_{o,c}\frac{1}{1+sT_{\sum s}}$$

where J and $B_{m}$ are the inertia and friction coefficient, respectively.

Third, the position closed-loop transfer function is derived, and the position control gain is determined using Equations (4).

(4) $$G_{c,p}=\frac{k_{pp}}{s+k_{pp}}$$

## 3. Results and Discussions

### 3.1. Results of Fuzzy Logic Gait Detection

If the inference result was consistent with the force information and the trend is reasonable, it could be compared with the calculated gaits to verify that the fuzzy logic gait inference result is consistent with the actual situation.

**Figure 14:**
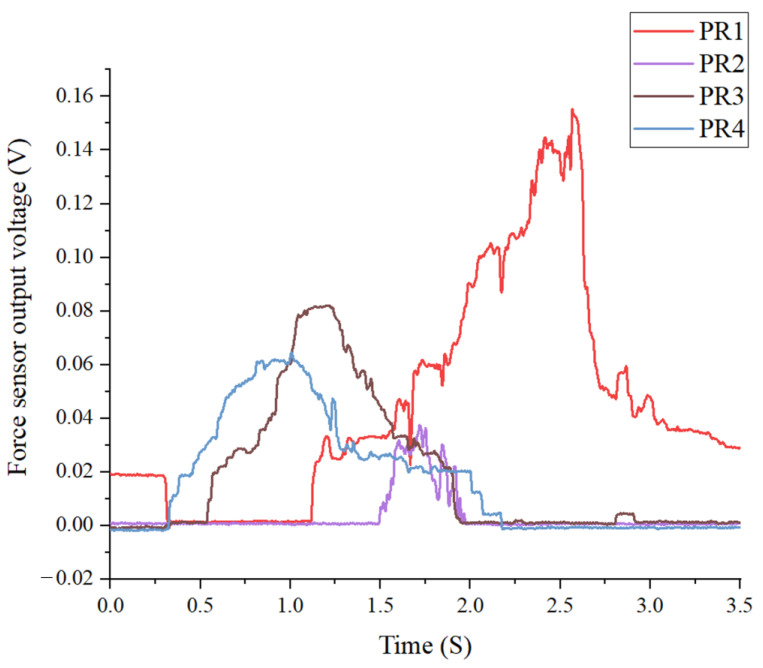
The signals of the piezoresistive sensors.

The data in Table 3 show that the horizontal axis represents the force received by the front sensors, while the vertical axis represents the force received by the rear sensors. Gait stage algebras showed inference results in the remaining blocks. PS and IC share the same belonging function model, so the system will first determine the value of the front and rear sensors after receiving the signal to distinguish them. Figure 14 shows the signals of the piezoresistive sensor array on the insole.

The sensor values for the front and rear groups ranged from [0, 0] to [50, 50], where minimal force was detected and primarily considered subtle noise, leading to the system inferring gait SW phase. As the values increased within [51, 0] to [75, 50], the fuzzy rules gradually transitioned the inference from SW to PS, resulting in an intermediate output of –1. Due to noticeable stress on the front sensors, this phase represented a transition between IC and PS. When the values reached [76, 0] to [200, 50], the system inferred a zero output, indicating the IC-PS transition state, and if the front sensor values exceeded 200, while the rear sensor values remained in this range, the phase was classified as PS. In contrast, for values within [0, 51] to [50, 75], the rear sensors recorded significant stress, causing the system to infer IC with an output of –1. If the values increased further from [0, 76] to [50, 200], the inference remained 0, confirming the IC phase, even if the force on the rear sensors exceeded 200, while the front sensors remained in this range. Finally, when both sensor values ranged between [51, 51] and [200, 200] or higher, the entire foot exerted significant pressure, causing the inference results to progress from 1 to 2, confirming the Mst. From a logical perspective, if front sensor values remain low while rear sensor values increased, the heel pressed harder, shifting the gait phase from –2 (SW) to 0 (IC). Conversely, if rear sensor values remained low while front sensor values increased, the toe pressed harder, transitioning the phase from –2 (SW) to 0 (PS). When both front and rear sensor values increased simultaneously, the entire foot stepped harder, causing a sequential progression in inference results from –2 to 2. Overall, the system’s inference logic followed a consistent and reasonable pattern, accurately reflecting gait phase transitions based on force distribution across the foot.

**Figure 15:**
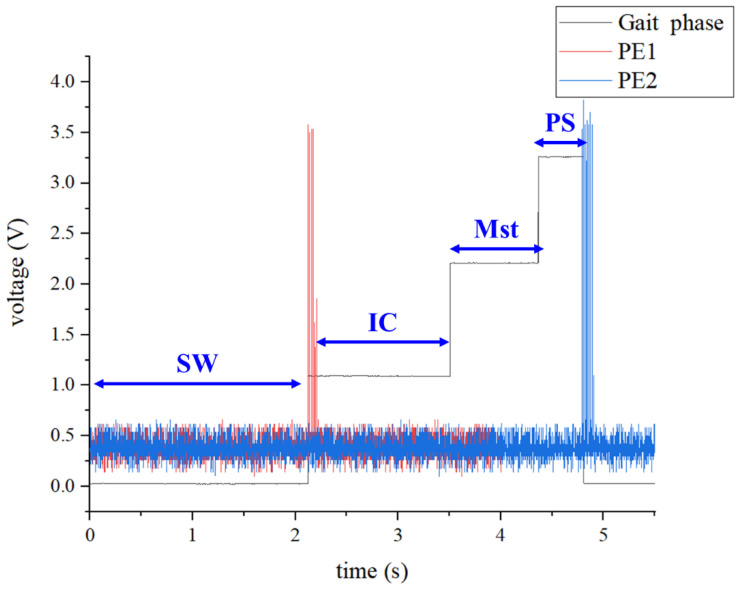
PVDF-based NFES sensors work with gait phase detection.

Figure 15 shows how the PVDF-based NFES sensors work with gait phase detection. The red line represents the PE1 on the front of the insole, which implies the start of the walking signal. The blue line represents the PE2 on the rear of the insole, which indicates the end of the walking signal. The black line means the gait cycle detection output signal, from 0 V to 1 V, for the SW stage, 1 V for the IC stage, 2 V for the MST stage, and 3 V for the PS stage. The result of the signal shows that piezoresistive and PVDF-based NFES sensors cooperate reasonably in detecting the exoskeleton user’s intent.

**Figure 16:**
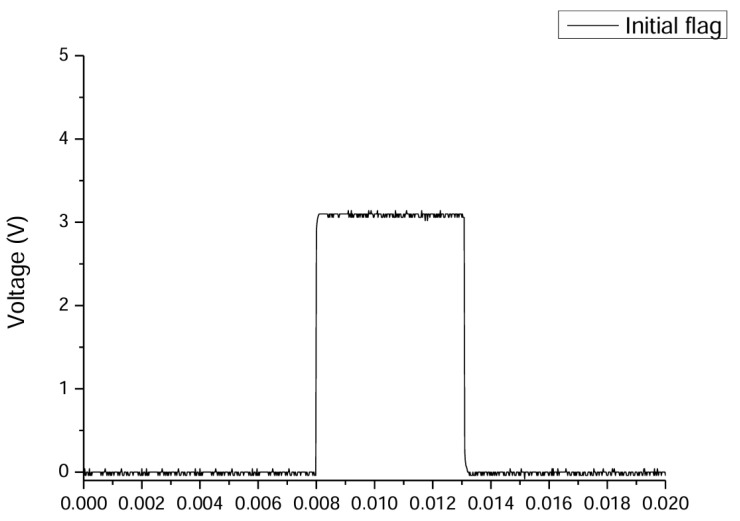
The total system communication and computation time is approximately 5.09 ms.

The results of the synchronization test for force feedback acquisition and completion of the follow-up response are shown in Figure 16. The overall system operation encompasses force feedback acquisition, ADC conversion, fuzzy logic gait determination, signal transmission to the motor controller for response execution, and response feedback, forming a complete operational loop. The results indicate that the system can complete one computation and communication cycle in approximately 5.09 ms, which includes approximately 4 ms for computations performed by the upper-level controller and around 1.09 ms for communication with the motor controller. This result demonstrates that the DSP has sufficient computational capability, enabling the system to respond in real time.

### 3.2. Comparison of Fuzzy Logic Gait Detection and Traditional Gait Detection

**Figure 17:**
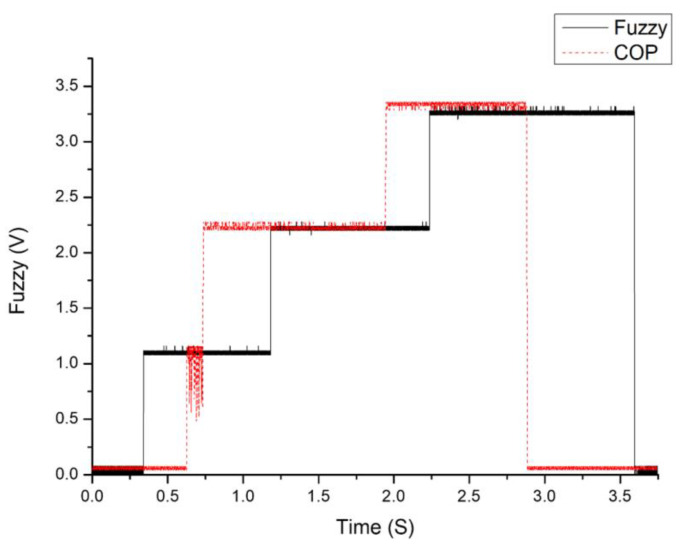
Comparison of fuzzy logic gait detection and traditional gait detection.

Figure 17 compares fuzzy logic-based and traditional gait detection methods using the center of gravity. The black line represents the fuzzy logic-based detection, while the red dashed line corresponds to the COG-based traditional detection. The *x*-axis denotes time in seconds, and the *y*-axis represents the detected gait phase voltage in volts. The phases are represented at distinct voltage levels: 0 V for the swing phase, 1 V for initial contact, 2 V for mid-stance, and 3 V for pre-swing. In this comparison, the fuzzy logic method demonstrates smoother transitions between gait phases with well-defined voltage plateaus, indicating consistent and accurate phase recognition. Conversely, the traditional COG detection shows fluctuating and less stable outputs, particularly during phase transitions. These fluctuations may lead to inaccuracies in determining the exact timing of transitions between gait phases, as seen by the inconsistent red dashed line during the transitions.

The superiority of fuzzy logic-based gait detection arises from its ability to simultaneously process and analyze multiple inputs using fuzzy membership functions and rules. By defining precise membership functions for ground reaction force distributions, the system can accurately identify gait phases even with variations in walking patterns or sensor noise. This adaptability ensures a reliable response across diverse conditions. In contrast, traditional COG-based methods rely on direct measurement and thresholds, making them more prone to errors in the presence of noise or dynamic variations in ground reaction forces. Such limitations result in less reliable and less adaptable gait phase detection. The fuzzy logic-based approach outperforms traditional detection methods by offering more stable and accurate phase identification. It is a more robust choice for lower-limb robotic orthoses applications and wearable rehabilitation devices. This improvement enhances the system’s ability to align exoskeleton movements with the user’s natural gait, providing smoother and more intuitive motion assistance.

### 3.3. Unloaded and Loaded Tests of the Robotic Orthosis

**Figure 18:**
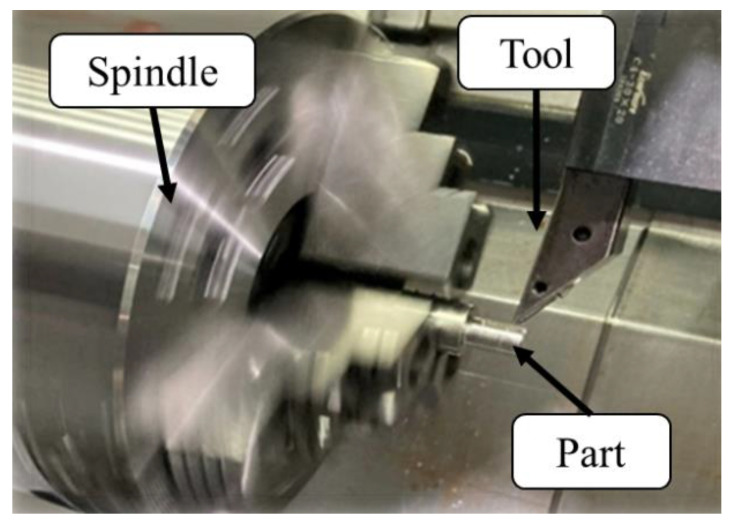
CNC machining.

**Figure 19:**
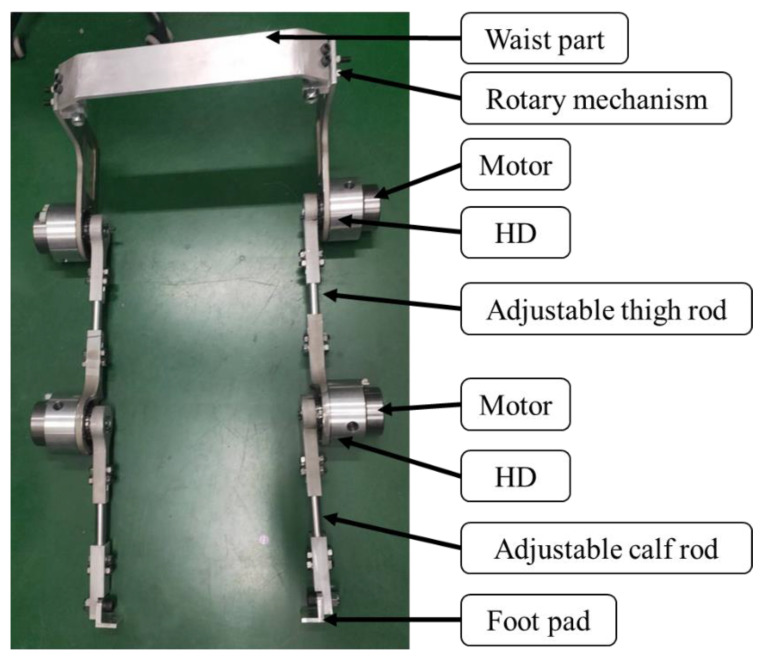
Assembled orthosis.

The authors processed the parts of the robotic orthosis in this study, as shown in Figure 18. After machining was complete, the parts were assembled to confirm fit and tolerances. As shown in Figure 19, the weight of the robotic orthosis assembled with four sets of motors and HDs was about 16 kg.

**Figure 20:**
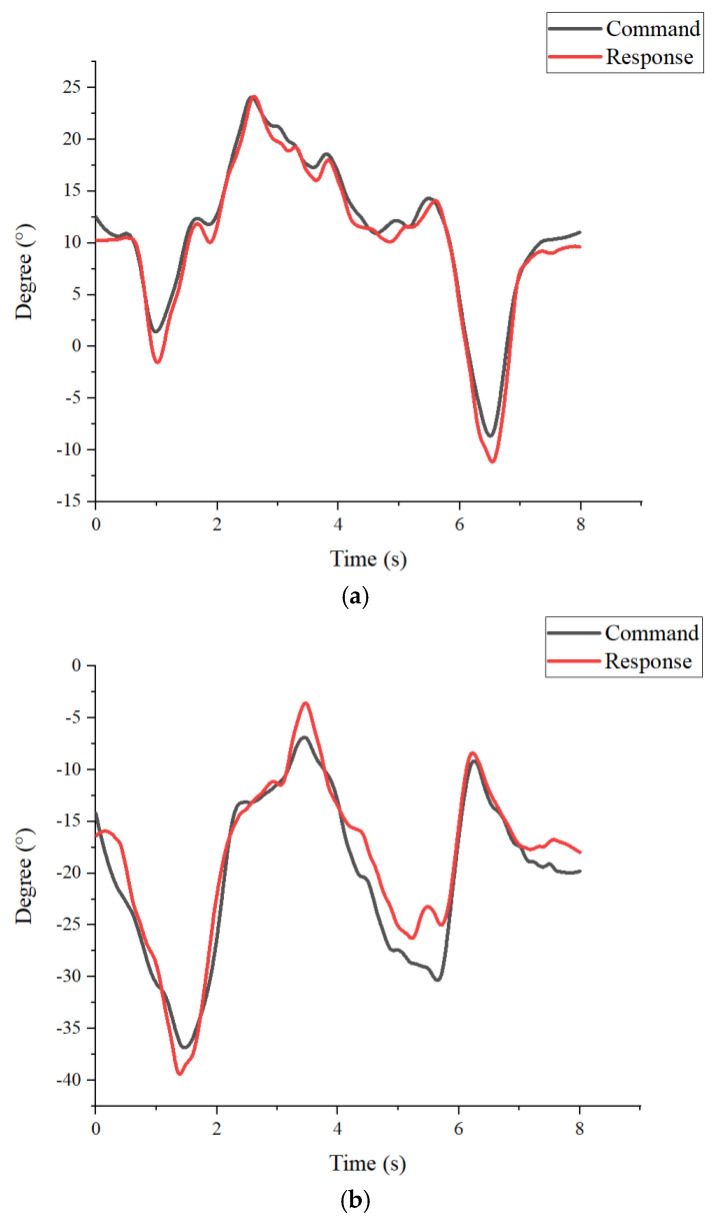
Results of 8-s walking cycle. (**a**) Tracking results of the hip (**b**) Tracking results of the knee.

**Figure 21:**
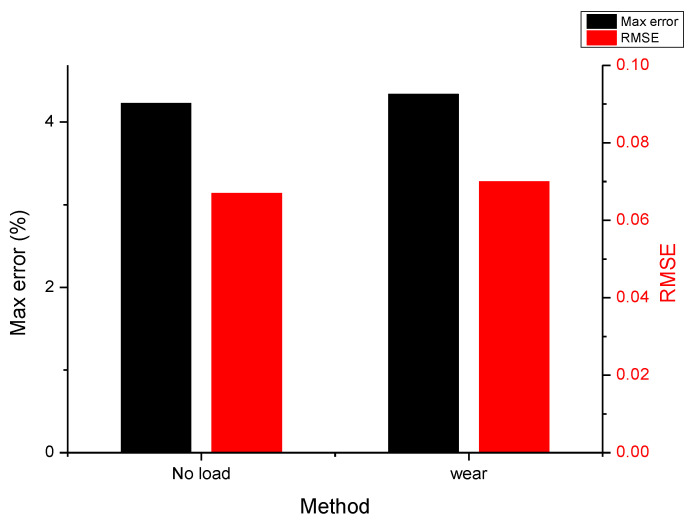
Comparison of the maximum error and RMSE.

Figure 20a,b show the response results of the lower-limb robotic orthosis system in the 8-s gait cycle test worn by a subject with a height of 173 cm and 68 kg. The system operated under a load of 68 kg, and the overall response was smooth and stable. The maximum error of the hip joint was about 4.34%, and the root mean squared error (RMSE) was 0.07. According to the test results, the system’s response performance and error were similar under various load conditions, as shown in Figure 21. The results showed that the overall operation of the lower-limb robotic orthosis was smooth under no-load conditions. The maximum error was about 4.23% at the hip joint, and RMSE was 0.067. Preliminary experimental results showed that the torque of the motors and the specifications of the entire system were suitable for motion control under both no-load and loaded (user-worn) conditions.

### 3.4. Experiments of the Motion Control

**Figure 22:**
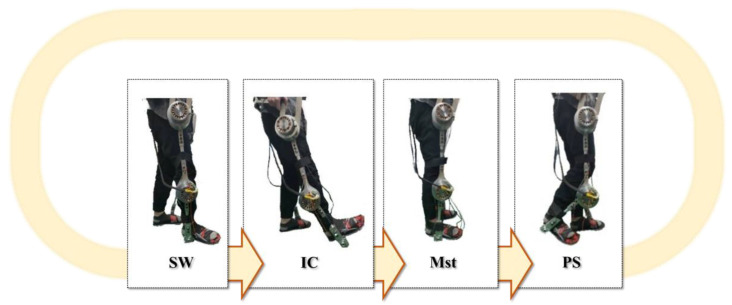
The action decomposition diagram of the robotic orthosis operation.

**Figure 23:**
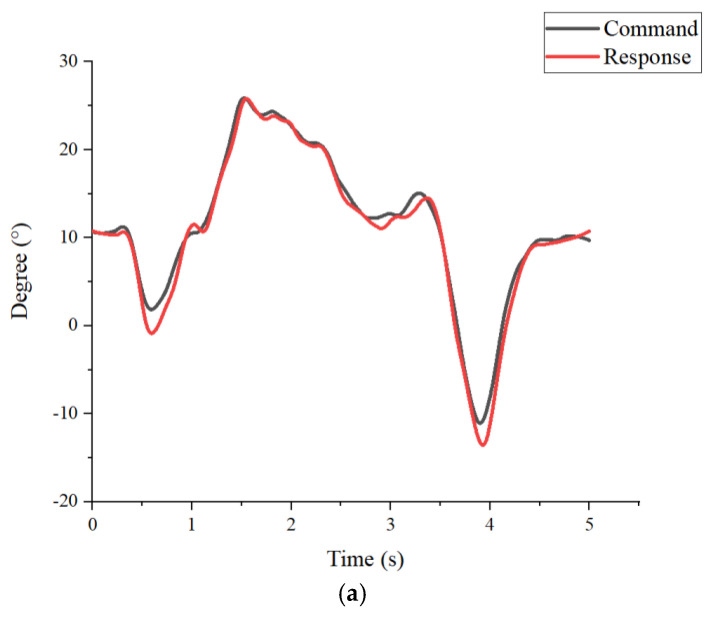

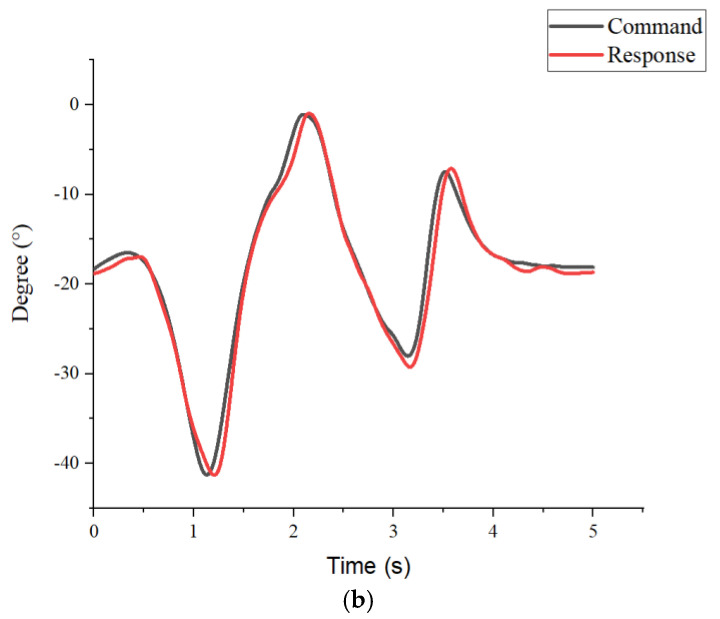
Results of the first experiment. (**a**) Tracking results of the hip (**b**) Tracking results of the knee.

**Figure 24:**
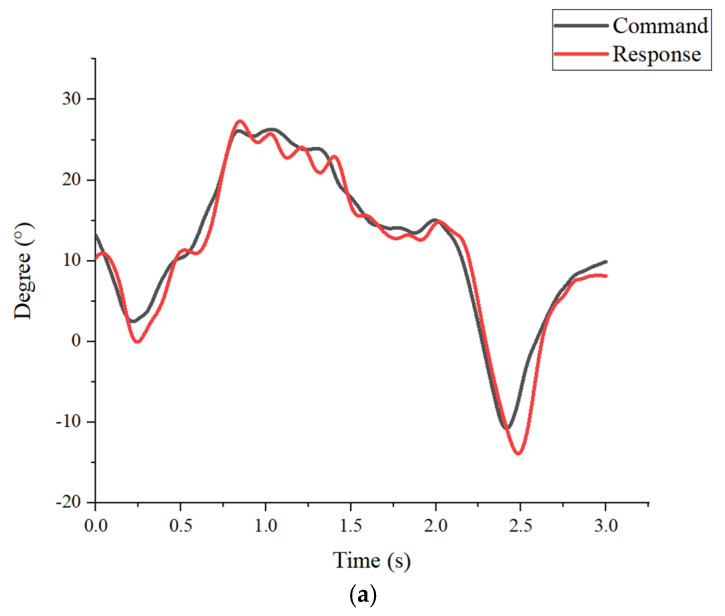

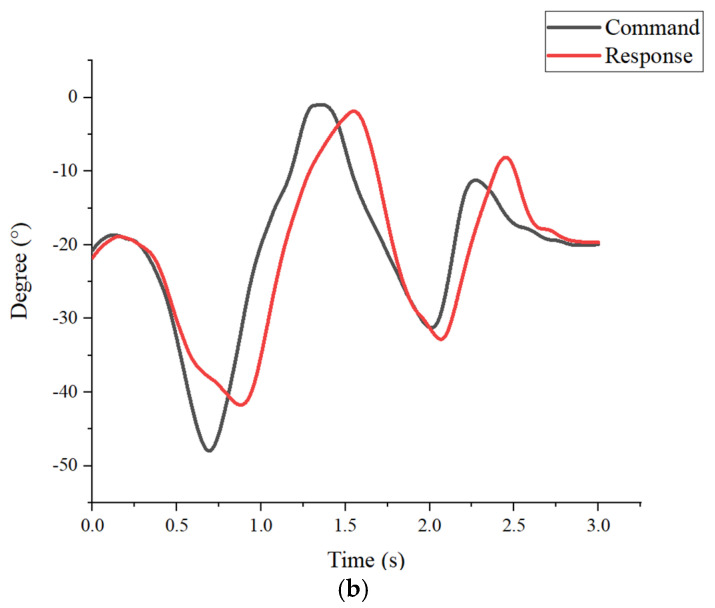
Results of the second experiment. (**a**) Tracking results of the hip (**b**) Tracking results of the knee.

The action decomposition diagram of the robotic orthosis operation is shown in Figure 22. Figure 23a,b show the results of the command signal and response signal of the hip and knee joints in the first experiment. The first subject has a height of 173 cm and a weight of 68 kg. Walking speed is about 5 s per cycle. The response trend had caught up, and the maximum error was at the knee joint, which was about 5.02%, and the RMSE was 1.63. Figure 24a,b show the results of the command signal and response signal of the hip and knee joints in the 3-s walking cycle. The second subject has a height of 167 cm and a weight of 77 kg. Walking speed is about 3 s per cycle. Its performance was close to the test, with a period of 5 s, indicating tolerances in the system. The maximum error was at the knee joint, about 6.79%, and the RMSE was 1.89. The results of the motion control are shown in Table 4.

## 4. Conclusions

The lower-limb exoskeleton robot system focuses on safety and lightweight construction. The components followed a symmetrical design, and incorporating a swing waist mechanism improved user comfort during walking. The system utilized fuzzy logic-based gait phase detection, while the NFES device was developed to produce electrospun fibers. These fibers were encapsulated into sensors and integrated with the exoskeleton to measure physiological signals. When pressure is applied to the sensor array on the insole, the system collects signals from the foot, enabling the detection of ground reaction forces. The system analyzed these forces using the fuzzy inference process to determine the gait phase. The master controller processed the fuzzy logic, simplifying the complex gait phase detection process. Experimental results demonstrated a sequential change in fuzzy inference outcomes, following a logical trend. The system relayed gait phase information through CAN Bus transmission to slave controllers, which then regulated the exoskeleton’s joint motors. During an 8-s gait cycle test, the maximum tracking error in the unloaded condition was 4.23%, with an RMSE of 0.067. When tested with a 68 kg subject, the maximum tracking error increased by 0.11%, and the RMSE increased by 0.003, indicating minimal deviation. These findings confirmed that the motor torque and system specifications effectively controlled motion under no-load and user-worn conditions. In faster motion control experiments, the maximum tracking errors for 5-s and 3-s gait cycles were 5.02% and 6.79%, respectively. The RMSE values for these cycles were 1.63 and 1.89, demonstrating accurate tracking performance. This exoskeleton system successfully detects human motion intention, is a wearable rehabilitation device, and opens new opportunities for medical treatment and mobility assistance.

## Figures and Tables

**Table 1:** Comparison of other exoskeletons. direct current (DC) motor, series elastic actuators (SEA), electromyographic (EMG) sensors, inertial measurement unit (IMU) sensors.

Reference	Purpose	Sensor	Actuator
[33]	power augmentation	pressure, torque, encoder	DC motor, cylinder
[34]	rehabilitation, assistive	pressure, IMU	DC motor
[35]	rehabilitation	torque, force	SEA, cylinder
[36]	rehabilitation	EMG, encoder	DC motor, SEA
[37]	assistive	pressure, force, camera	SEA
[38]	assistive	force	brushless, cable-driven
This work	rehabilitation, assistive	force, encoder	brushless

**Table 2:** Fuzzy rules.

Fuzzy Rules	
Conditions	Gait Phase
The front group and rear group are small	SW
The front group is small, and the rear group is medium	IC
The front group is small, and the rear group is large	IC
The front group is medium, and the rear group is small	PS
The front group and the rear group are medium	Mst
The front group is medium, and the rear group is large	Mst
The front group is large, and the rear group is small	PS
The front group is large, and the rear group is medium	Mst
The front group and rear group are large	Mst

**Table 3:** Results of fuzzy logic-based gait phase detection.

		Front	PS	PS	PS	PS	PS	PS	PS	PS	
	SW	0	25	50	75	100	125	150	175	200	
Rear	0	−2	−2	−2	−1	0	0	0	0	0	
IC	25	−2	−2	−2	−1	0	0	0	0	0	
IC	50	−2	−2	−2	−1	0	0	0	0	0	
IC	75	−1	−1	−1	1	1	1	1	1	1	
IC	100	0	0	0	1	2	2	2	2	2	Mst
IC	125	0	0	0	1	2	2	2	2	2	Mst
IC	150	0	0	0	1	2	2	2	2	2	Mst
IC	175	0	0	0	1	2	2	2	2	2	Mst
IC	200	0	0	0	1	2	2	2	2	2	Mst
						Mst	Mst	Mst	Mst	Mst	Mst

**Table 4:** Results of the motion control.

Walking Cycle	Maximum Error	RMSE
3 s	6.79%	1.89
5 s	5.02%	1.63

## Data Availability

The raw data supporting the conclusions of this article will be made available by the authors upon request.
